# Genome-Scale Data Call for a Taxonomic Rearrangement of *Geodermatophilaceae*

**DOI:** 10.3389/fmicb.2017.02501

**Published:** 2017-12-19

**Authors:** Maria del Carmen Montero-Calasanz, Jan P. Meier-Kolthoff, Dao-Feng Zhang, Adnan Yaramis, Manfred Rohde, Tanja Woyke, Nikos C. Kyrpides, Peter Schumann, Wen-Jun Li, Markus Göker

**Affiliations:** ^1^School of Biology, Newcastle University, Newcastle upon Tyne, United Kingdom; ^2^Leibniz Institute, German Collection of Microorganisms and Cell Cultures, Braunschweig, Germany; ^3^State Key Laboratory of Biocontrol and Guangdong Provincial Key Laboratory of Plant Resources, School of Life Sciences, Sun Yat-sen University, Guangzhou, China; ^4^Department of Biotechnology, Middle East Technical University, Ankara, Turkey; ^5^Central Facility for Microscopy, Helmholtz Centre for Infection Research, Braunschweig, Germany; ^6^Department of Energy, Joint Genome Institute, Walnut Creek, CA, United States

**Keywords:** *Klenkia*, *Geodermatophilus*, *Modestobacter*, Blastococcus, GBDP, GGDC, phylogenetic systematics, polyphasic taxonomy

## Abstract

*Geodermatophilaceae* (order *Geodermatophilales*, class *Actinobacteria*) form a comparatively isolated family within the phylum *Actinobacteria* and harbor many strains adapted to extreme ecological niches and tolerant against reactive oxygen species. Clarifying the evolutionary history of *Geodermatophilaceae* was so far mainly hampered by the insufficient resolution of the main phylogenetic marker in use, the 16S rRNA gene. In conjunction with the taxonomic characterisation of a motile and aerobic strain, designated YIM M13156^T^ and phylogenetically located within the family, we here carried out a phylogenetic analysis of the genome sequences now available for the type strains of *Geodermatophilaceae* and re-analyzed the previously assembled phenotypic data. The results indicated that the largest genus, *Geodermatophilus*, is not monophyletic, hence the arrangement of the genera of *Geodermatophilaceae* must be reconsidered. Taxonomic markers such as polar lipids and fatty-acids profile, cellular features and temperature ranges are indeed heterogeneous within *Geodermatophilus*. In contrast to previous studies, we also address which of these features can be interpreted as apomorphies of which taxon, according to the principles of phylogenetic systematics. We thus propose a novel genus, *Klenkia*, with the type species *Klenkia marina* sp. nov. and harboring four species formerly assigned to *Geodermatophilus, G. brasiliensis, G. soli, G. taihuensis*, and *G. terrae*. Emended descriptions of all species of *Geodermatophilaceae* are provided for which type-strain genome sequences are publicly available. Our study again demonstrates that the principles of phylogenetic systematics can and should guide the interpretation of both genomic and phenotypic data.

## Introduction

The order *Geodermatophilales* (Sen et al., 2014) comprises the sole family *Geodermatophilaceae*, which was initially proposed by Normand et al. (1996), although no type genus was designated at that time, confirmed later by Stackebrandt et al. (1997), formally described by Normand (2006) and later emended by Zhi et al. (2009). The family accommodates the genera *Blastococcus* (Ahrens and Moll, 1970; Skerman et al., 1980; Hezbri et al., 2016b), *Modestobacter* (Mevs et al., 2000) and the type genus *Geodermatophilus* (Luedemann, 1968; Skerman et al., 1980).

*Geodermatophilus* was historically poorly studied due to difficulties in culturing novel isolates (Urzì et al., 2004). Overcoming those technical difficulties, the number of validly named species within the genus dramatically increased from a single species, *G. obscurus*, in 2011 to twenty-one species at the time of writing (Parte, 2014). The number of *Geodermatophilus* isolates is expected to continue to raise in coming years, as indicated by metagenomics studies carried out in arid and hyper-arid habitats (Neilson et al., 2012; Giongo et al., 2013). Species belonging to the genus are indeed mainly isolated from arid soils and characterized by tolerance against oxidative stress (Gtari et al., 2012; Montero-Calasanz et al., 2013a, 2014, 2015; Hezbri et al., 2015a, 2016a) although some isolates from rhizospheric soils and lake sediments have also been classified within the genus.

In the post-genomic era, the integration of genomic information in microbial systematics (Klenk and Göker, 2010) in addition to physiological and chemotaxonomic parameters as taxonomic criteria is strongly suggested for classifying prokaryotes (Ramasamy et al., 2014). This particularly holds in groups such as *Geodermatophilaceae*, which are only incompletely resolved in phylogenies inferred from the most commonly applied marker gene, the 16S rRNA gene. Nevertheless, except for the genome sequences generated within our project only the genome (Ivanova et al., 2010) and proteome sequence of *G. obscurus* (Sghaier et al., 2016) were publicly available.

Based on phylogenies inferred from genome-scale data and on a re-interpretation of the available phenotypic evidence according to the principles of phylogenetic systematics (Hennig, 1965; Wiley and Lieberman, 2011), this study introduces the new genus *Klenkia* into *Geodermatophilaceae*, whose type species is *Klenkia marina* sp. nov. Accordingly, we also propose the reclassification of *G. brasiliensis* as *Klenkia brasiliensis* comb. nov., *G. soli* as *K. soli* comb. nov., *G. taihuensis* as *K. taihuensis* comb. nov. and *G. terrae* as *K. terrae* comb. nov., as well as emended descriptions within *Geodermatophilus*.

## Materials and Methods

### Isolation

Strain YIM M13156^T^ was isolated from a sample collected from the South China Sea (119° 31.949E, 18° 2.114 N), and was obtained using the serial dilution technique. Sediment sample (1 g) was added to 9 ml sterile distilled water and mixed by vortexing. A 10-fold dilution of this soil suspension was prepared in sterilized distilled water, and 0.1 ml was spread on Fucose-proline agar medium [fucose 5 g; proline 1 g; (NH_4_)_2_SO_4_ 1 g; NaCl 1 g;CaCl_2_ 2 g; K_2_HPO_4_ 1 g; B-Vitamin trace (0.5 mg each of thiamine-HCl (B_1_), riboflavin, Niacin, pyridoxin, Ca-pantothenate, inositol, *p*-aminobenzoic acid, and 0.25 mg of biotin); sea salt 30 g; agar 20 g; pH 7.2; distilled water 1 liter]. The plate was then incubated at 28°C for 30 days.

### Phenotypic Analysis

#### Morphological and Physiological Tests

Morphological characteristics of strain YIM M13156^T^ were determined on GYM *Streptomyces* medium at 28°C. Colony features were observed at 4 and 15 days under a stereo microscope according to Pelczar (1957). Exponentially growing bacterial cultures were observed with an optical microscope (Zeiss AxioScope A1) with a 1000-fold magnification and phase-contrast illumination. Gram reaction was performed using the KOH test described by Gregersen (1978). Oxidase activity was analyzed using filter-paper disks (Sartorius grade 388) impregnated with 1% solution of *N,N,N′,N′*-tetramethyl-*p*-phenylenediamine (Sigma–Aldrich); a positive test was defined by the development of a blue-purple color after applying biomass to the filter paper. Catalase activity was determined based on formation of bubbles following the addition of 1 drop of 3% H_2_O_2_. Growth rates were determined on plates of GYM *Streptomyces* medium for temperatures from 10°C to 50°C at 5°C increments and for pH values from 4.0 to 12.5 (in increments of 0.5 pH units) on modified ISP2 medium by adding NaOH or HCl, respectively, since the use of a buffer system inhibited growth of the strain. The oxidation of carbon compounds was tested at 28°C using GEN III Microplates in an Omnilog device (BIOLOG Inc., Hayward, CA, United States) in comparison with the reference strains *G. brasiliensis* DSM 44526^T^, *G. soli* DSM 45843^T^, *G. taihuensis* DSM 45962^T^, *G. terrae* DSM 45844^T^ in parallel assays. The GEN III Microplates were inoculated with cells suspended in a viscous inoculating fluid (IF C) provided by the manufacturer at a cell density of 94% T for *G. brasiliensis* DSM 44526^T^, 83% T for *G. soli* DSM 45843^T^ and *G. terrae* DSM 45844^T^and 90% T for *G. taihuensis* DSM 45962^T^ and for the strain YIM M13156^T^. Respiration rates were measured yielding a total running time of 5 days in Phenotype Microarray mode. Each strain was studied in two independent technical replicates. Data were exported and analyzed using the *opm* v.1.0.6 package (Vaas et al., 2012, 2013) for the *R* statistical environment (R Core Team, 2017). Reactions with a distinct behavior between the two replicates were regarded as ambiguous.

#### Chemotaxonomic Tests

Whole-cell amino acids and sugars were prepared according to Lechevalier and Lechevalier (1970), followed by thin-layer chromatography (TLC) analysis (Staneck and Roberts, 1974). Polar lipids were extracted, separated by two-dimensional TLC and identified according to procedures outlined by Minni et al. (1984) with modifications proposed by Kroppenstedt and Goodfellow (2006). For identification the presumed OH-PE spots were manually scraped off from unstained TLC plates, extracted with methanol and evaporated to dryness. The extracts were then dissolved in Reagent 3 (fatty acid extraction) and analyzed by MIDI System. Menaquinones (MK) were extracted from freeze-dried cell material using methanol as described by Collins (1985) and analyzed by high-performance liquid chromatography (HPLC) (Kroppenstedt, 1982). The extraction and analysis of cellular fatty acids was carried out in two independent repetitions from biomass grown on GYM agar plates held at 28°C for 4 days and harvested always from the same sector (the last quadrant streak). Analysis was conducted using the Microbial Identification System (MIDI) Sherlock Version 4.5 (method TSBA40, ACTIN6 database) as described by Sasser (1990). The annotation of the fatty acids in the ACTIN6 peak naming table is consistent with IUPAC nomenclature. Fatty-acid patterns were visualized as a heatmap using the lipid extension of the *opm* package (Vaas et al., 2013) and clustered using the *pvclust* v.1.2.2 package (Suzuki and Shimodaira, 2006) for the *R* statistical environment. Quantitative analysis of the fatty acids used logit-transformed percentages throughout (after setting zero values to the lowest non-zero number) because proportion data are expected to vary stronger around 50% than close to 0 or 100% (Crawley, 2007). All chemotaxonomic analyses were conducted under standardized conditions for strain YIM M13156^T^ and the type strains listed above.

Except for the separately stored MIDI measurements, all phenotypic characters were collected in a standardized tabular format that constitutes Supplementary Table S1. Custom scripts developed at DSMZ allow for extracting such data in ways suitable for subsequent phylogenetic or other analysis. For determining features specific for predetermined groups of interest, such as new taxa suggested by phylogenetic analysis, the *randomForest* function from the eponymous *R* package v. 4.6-12 (Breiman, 2001) was applied in classification mode under default settings except for increased values of *ntree* (50,000) and *mtry* (half the number of analyzed features).

### Sequence Analysis

For 16S rRNA gene sequencing, genomic DNA extraction, PCR-mediated amplification of the 16S rRNA gene and purification of the PCR product was carried out as described by Rainey et al. (1996). For genome sequencing, the strain YIM M13156^T^ and sixteen species with validly published *Geodermatophilus* names were cultivated in GYM *Streptomyces* broth at 28°C. The project information is available through the Genomes Online Database (Mukherjee et al., 2017). The draft genomes were generated at the DOE Joint Genome Institute (JGI) as part of Genomic Encyclopedia of Archaeal and Bacterial Type Strains, Phase II (KMG-II): from individual species to whole genera (Kyrpides et al., 2014) following the same protocol as in Nouioui et al. (2017). All genomes were annotated using the DOE-JGI annotation pipeline (Huntemann et al., 2015; Chen et al., 2016) and released through the Integrated Microbial Genomes system (Chen et al., 2017).

Phylogenetic analysis of the 16S rRNA gene sequences from the type strains of all species with effectively published names in *Geodermatophilaceae*, as well as the genome-sequenced strains *Cryptosporangium arvum* DSM 44712^T^ and *Sporichthya polymorpha* DSM 43042^T^ for use as outgroup, was conducted as previously described (Göker et al., 2011; Montero-Calasanz et al., 2014b). Pairwise 16S rRNA gene similarities were calculated as recommended by Meier-Kolthoff et al. (2013b) to determine strains with ≥99.0% similarity, between which (digital) DNA:DNA hybridization experiments should be conducted. Genome-scale phylogenies were inferred from the available *Geodermatophilaceae* (and outgroup) whole proteome sequences using the high-throughput version (Meier-Kolthoff et al., 2014a) of the genome BLAST Distance Phylogeny (GBDP) approach (Auch et al., 2010) in conjunction with FastME (Lefort et al., 2015) as described earlier (Hahnke et al., 2016). An additional FastME tree was inferred without the two outgroup genomes to detect potential long-branch attraction to the outgroup, a process called long-branch extraction (Siddall and Whiting, 1999). Additionally, the validity of the rooting was tested by re-estimating the root (Simon et al., 2017) using least-squares dating (To et al., 2015). The GBDP tree restricted to the well-supported branches (≥95% pseudo-bootstrap support) was used as a backbone constraint in a further 16S rRNA gene analysis to integrate information from genome-scale data (Hahnke et al., 2016). Digital DNA:DNA hybridisations were conducted using the recommended settings of the Genome-To-Genome Distance Calculator (GGDC) version 2.1 (Meier-Kolthoff et al., 2013a). The G+C content was calculated from the genome sequences as described by Meier-Kolthoff et al. (2014b).

## Results

### Sequence Analysis

**FIGURE 1:**
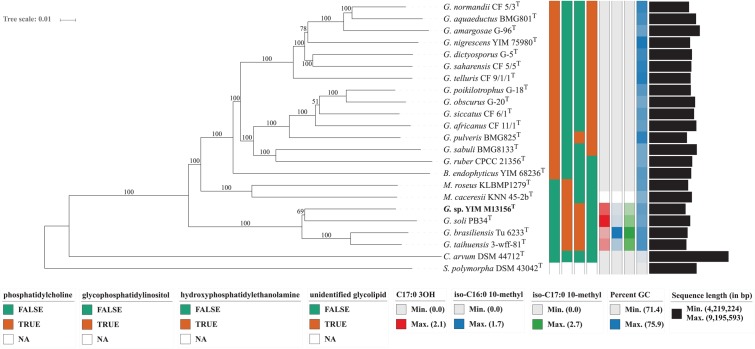
Phylogenomic tree inferred with GBDP. The tree was inferred with FastME from GBDP distances calculated from whole proteomes. The numbers above branches are GBDP pseudo-bootstrap support values from 100 replications. Tip colors indicate chemotaxonomic characters that provide apomorphies for groups of interest, genome sizes and the exact G+C content as calculated from the genome sequences (see the embedded legend for details). *B., Blastococcus*; *C., Cryptosporangium; G., Geodermatophilus; M., Modestobacter; S., Sporichthya*; NA, not applicable.

**FIGURE 2:**
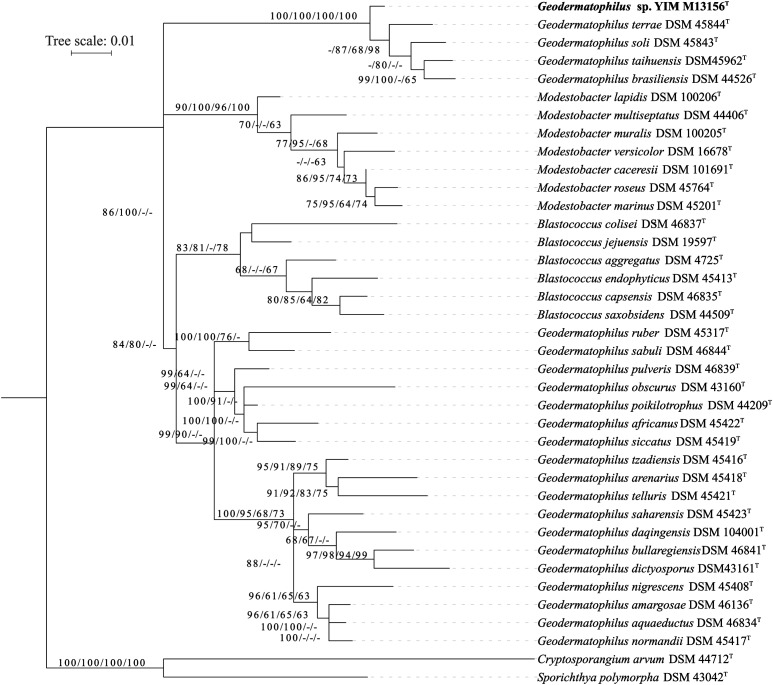
Maximum likelihood phylogenetic tree inferred from 16S rRNA gene sequences, showing the phylogenetic position of the strain YIM M13156^T^ relative to the type strains within *Geodermatophilaceae*. The branches are scaled in terms of the expected number of substitutions per site (see size bar). Support values from maximum-likelihood constrained (first), maximum-parsimony constrained (second), maximum-likelihood unconstrained (third) and maximum-parsimony unconstrained (fourth) bootstrapping are shown above the branches if equal to or larger than 60%.

The phylogenetic tree based on the whole proteomes of the sequenced type strains placed, with maximum support, the group formed by the strain YIM M13156^T^, *G. brasiliensis* DSM 44526^T^, *G. soli* DSM 45843^T^ and *G. taihuensis* DSM 45962^T^ as a sister-group of all other *Geodermatophilaceae* genera (**Figure 1**). Hence *Geodermatophilus* is obviously non-monophyletic according to this tree, as *Modestobacter* as well as *Blastococcus* appeared as more closely related to core *Geodermatophilus* (including the type species *G. obscurus*) than the four deviating *Geodermatophilus* strains. When *C. arvum* and *S. polymorpha* were removed, the resulting unrooted topologically was identical to the (unrooted) ingroup topology shown in **Figure 1**. That is, it was impossible to root the reduced tree in a way that made *Geodermatophilus* monophyletic, indicating that the non-monophyly of *Geodermatophilus* was not caused by a long-branch attraction artifact. Least-squares dating confirmed the rooting in both the full and the reduced tree. As expected, the unconstrained phylogenetic tree based on 16S rRNA gene sequences was not well resolved at its backbone but when applying the constraint derived from the GBDP tree, *Modestobacter*, *Blastoccocus* and the two distinct groups of *Geodermatophilus* appeared as monophyletic (**Figure 2**); support for their monophyly was strong except in the case of *Blastococcus*. Moderate to strong support was obtained for *Modestobacter* and *Blastoccocus* being more closely related to core *Geodermatophilus* than the group formed by the strain YIM M13156^T^ as well as the species *G. brasiliensis, G. soli, G. taihuensis* and *G. terrae*.

Within this clade, the 16S rRNA gene sequence of strain YIM M13156^T^ showed similarities ≥ 99.0% with the type strains of *G. soli* (99.1%) and *G. brasiliensis* (99.0%) only; those with *G. terrae* (98.9%) and *G. taihuensis* (98.0%) were lower. Digital DNA:DNA hybridisations between strain YIM M13156^T^ and *G. soli* DSM 45843^T^ and *G. brasiliensis* DSM 44526^T^ resulted in 29.8 and 29.6% similarity, respectively, clearly below the 70% threshold recommended by Wayne et al. (1987) to confirm the species status of novel strains. Additional digital DNA:DNA hybridisations were not conducted, based on the observation of Meier-Kolthoff et al. (2013b) that an *Actinobacteria*-specific 16S rRNA threshold of 99.0% yielded a maximum probability of error of only 1% to obtain DNA:DNA hybridization values ≥ 70%.

The genome size range found in the sequenced type strains varied between 4.2 Mbp for the strain YIM M13156^T^ and 5.9 Mbp for *G. amargosae* DSM 46136^T^ (**Figure 1**). The genomic G+C content of strain YIM M13156^T^ was 74.4%. For the other genomes it ranged between 74.0% for *G. obscurus, G. ruber* and *G. sabuli* and 75.9% for *G. nigrescens* (**Figure 1**). Because G+C content values do not differ more than 1% within bacterial species (Meier-Kolthoff et al., 2014b), stronger deviations are due to artifacts in conventionally determined G+C content values. Hence, we accordingly propose to emend the species descriptions of those species for which we observed a deviation from published G+C content values > 1%.

### Phenotypic Analysis

#### Morphology and Physiology

Strain YIM M13156^T^ showed motile, rod-shaped and Gram-positive cells. These observations are in line with those described by Jin et al. (2013), Qu et al. (2013), and Bertazzo et al. (2014) for *G. soli* and *G. terrae*, *G. taihuensis*, and *G. brasiliensis*, respectively. In contrast to these four species, neither high aggregates forming multilocular sporangia nor zoospores were identified in YIM M13156^T^ cultures (Supplementary Table S1). Colonies were pink-colored, convex, circular and opaque with a smooth surface and an entire margin, an appearance similar to other *Geodermatophilus* species when cultivated under the same growth conditions. Cell growth ranged from 20 to 35°C (optimal growth temperature 25–30°C) and from pH 6.0 to 8.5 (optimal pH 6.5–8.0). Results from phenotype microarray analysis are shown as a heatmap in the Supplementary Material (Supplementary Figure S1) in comparison to the type strains of the four most closely related species. Differences between species were much more pronounced than between replicates. A summary of selected phenotypic characteristics is presented in **Table 1** (for an overview of phenotypic profiles in *Geodermatophilus* see Supplementary Table S1).

**Table 1:** Phenotypic characteristics of strain YIM M13156^T^ in comparison to those of the type strains of the most closely related *Geodermatophilus* species.

Characteristics	1	2	3	4	5
Motility	+	-	+	+	+
Oxidation of:					
Stachyose	-	-	+	+/-	-
α-D-Lactose	-	+	+	+/-	+
D-Melibiose	-	+/-	+	+	-
*N*-Acetyl-D-Galactosamine	-	+	-	-	-
D-Mannose	+	+	+	-	+
L-Rhamnose	--	+	+	-	+
L-Aspartic acid	-	-	-	-	+
Quinic acid	-	+	+	+	+
L-Lactic acid	-	-	+	-	+
Citric acid	+	-	+	-	-
α-Keto-Glutaric acid	+	-	-	-	+
D-Malic acid	+	-	+	-	+
L-Malic acid	+	-	+	-	+
Polar lipids	DPG, PE, OH-PE, PI, GPI	DPG, PE, OH-PE, PI, GPI	DPG, PG, PE, OH-PE, PI, GPI, GPL	DPG, PG, PE, OH-PE, PI, GPI	DPG, PG, PE, OH-PE, PI, GPI
Sugars	Rham, Rib, Man, Gluc, US	Rib, Man, Gluc, Gal	Rib, Man, Gluc, Gal	Rib, Man, Gluc, Gal	Rib, Man, Gluc, Gal
Menaquinones^a#^	MK-9(H_4_), MK-9(H_0_)	MK-9(H_4_), MK-9(H_2_), MK-9(H_0_), MK-10(H_4_)	MK-9(H_4_), MK-9(H_2_), MK-9(H_0_)	MK-9(H_4_), MK-9(H_2_), MK-9(H_0_), MK-8(H_4_)	MK-9(H_4_), MK-9(H_2_), MK-9(H_0_), MK-10(H_4_)
Fatty acids^b#^	iso-C_16:0_, C_17:1_*ω*8c, iso-C_15:0_	iso-C_15:0,_ iso-C_16:0_	iso-C_15:0,_ iso-C_16:0,_ iso-C_17:0_	iso-C_15:0,_ iso-C_16:0_	iso-C_16:0,_ iso-C_15:0,_ C_18:1_*ω*9c

*Strains: 1, strain YIM M13156^*T*^; 2, *G. brasiliensis* DSM 44526^*T*^; 3, *G. soli* DSM 45843^*T*^; 4, *G. taihuensis* DSM 45962^*T*^; 5, *G. terrae* DSM 45844^*T*^. All data are from this study. +, positive reaction; -, negative reaction; +/-, ambiguous; DPG, diphosphatidylglycerol; PE, phosphatidylethanolamine; PE-OH, hydroxy-phosphatidylethanolamine; PG, phosphatidylglycerol; PI, phosphatidylinositol; GPL, unidentified glycophospholipid; APL, unidentified amino-phospholipid; Rham, rhamnose; Rib, ribose; Man, mannose; Gluc, glucose; US, unidentified sugar; MK, menaquinones; iso-, iso-branched.*^a^*only components making up ≥ 1% peak area ratio are shown;*^b^*only components making up ≥ 10% peak area ratio are shown; ^#^, the components are listed in decreasing order of quantity*.

The *randomForest* analysis indicated that among the previously mentioned phenotypic features the cell shape well discriminates between the group formed by strain YIM M13156^T^, *G. brasiliensis, G. soli, G. taihuensis* and *G. terrae*, which produce rods, on the one hand and core *Geodermatophilus*, which is characterized by pleomorphic cells (and seldom cocci), on the other hand (Supplementary Figure S2).

#### Chemotaxonomy

Analysis of whole-cell components revealed the presence of *meso*-diaminopimelic acid (Cell-wall type III), which is consistent with the other representatives of *Geodermatophilaceae* (Normand and Benson, 2012).

Strain YIM M13156^T^ displayed primarily menaquinone MK-9(H_4_) (52.7%), in agreement with values reported for *Geodermatophilaceae* (Normand, 2006), and MK-9(H_0_) (39.2%). The presence of a significant amount of MK-9(H_0_) was already mentioned in the original descriptions of *G. brasiliensis*, *G. soli, G. taihuensis*, and *G. terrae*, but also in other *Geodermatophilus* species (Montero-Calasanz et al., 2015). In contrast to the already described profiles of isoprenologs of *G. soli, G. taihuensis* and *G. terrae*, traces of MK-9(H_2_) (6.6, 1.5, and 2.8%, respectively) were now additionally identified in those type strains. MK-8(H_4_) (5.7%) and MK-10(H_4_) (3.4%) were also detected in the patterns of *G. taihuensis* DSM 45962^T^ and *G. terrae* DSM 45844^T^, respectively.

**FIGURE 3:**
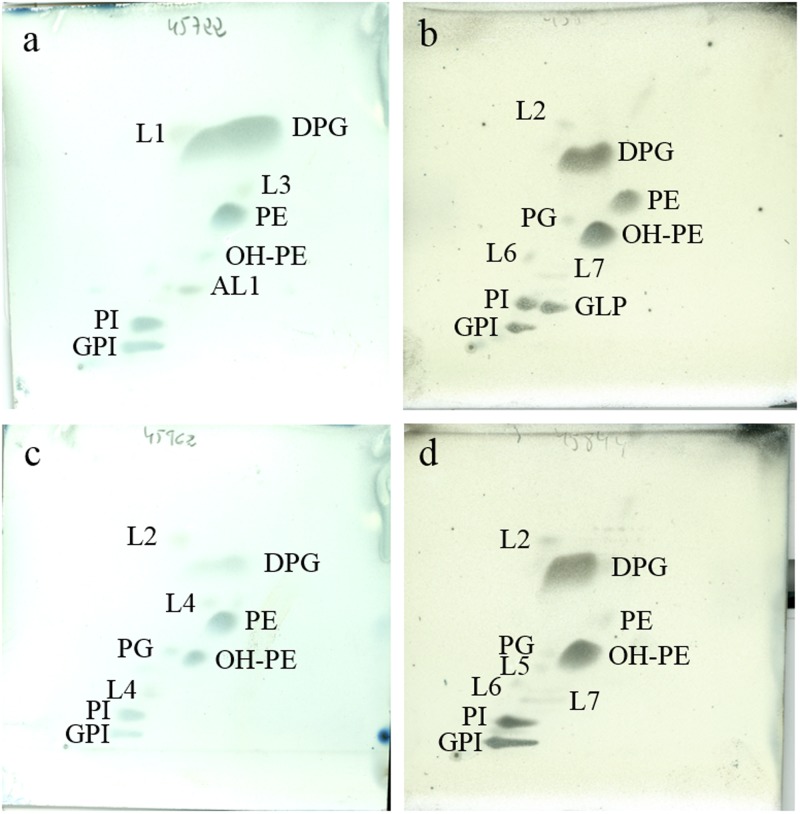
Polar lipids profile of strain YIM M13156^T^ **(a)**, *G. soli* DSM 45843^T^ **(b)**, *G. taihuensis* DSM 45962^T^ **(c)** and *G. terrae* DSM 45844^T^ **(d)** after separation by two-dimensional TLC using the solvents chloroform:methanol:water (65:25:4; v:v:v) in the first dimension and chloroform:methanol:acetic acid:water (80:12:15:4; v:v:v:v) in the second one. Plates were sprayed with molybdatophosphoric acid (3.5%; Merck^TM^) for detection of the total polar lipids. DPG, diphosphatidylglycerol; PG, phosphatidylglycerol; PE, phosphatidylethanolamine; OH-PE, hydroxyphosphatidylethanolamine; PI, phosphatidylinositol; GPI, glycophosphatidylinositol; GPL, unidentified glycophospholipid; AL, aminolipid; L1-7, unidentified lipids. All data are from this study.

The polar lipid pattern of strain YIM M13156^T^ consisted of diphosphatidylglycerol (DPG), phosphatidylethanolamine (PE), phosphatidylinositol (PI), glycophosphatidylinositol (GPI), an unidentified aminolipid (AL) and traces of hydroxyphosphatidylethanolamine (OH-PE) (**Figure 3a**). It is in accordance with patterns obtained for the closely related species investigated in this study (**Figures 3b–d**) and the phospholipid pattern revealed by Bertazzo et al. (2014) for *G. brasiliensis*. The *randomForest* analysis detected the lack of phosphatidylcholine and the presence of glycophosphatidylinositol as excellently predictive of the prospective new genus, whereas the absence of phosphatidylglycerol was also observed in other *Geodermatophilus* species (Bertazzo et al., 2014; Montero-Calasanz et al., 2014b, 2015; Hezbri et al., 2015a,b,c, 2016a). The unambiguous presence of hydroxyphosphatidylethanolamine is a slightly less relevant taxonomic marker for the prospective new genus; among core *Geodermatophilus* it was only detected in the polar lipid profile of *G. pulveris* DSM 45839^T^ by Hezbri et al. (2016a).

Even though both absence of phosphatidylcholine and presence of glycophosphatidylinositol were also observed in the polar-lipid profiles of some *Modestobacter* species (Montero-Calasanz et al., in preparation), the additional presence of hydroxyphosphatidylethanolamine forms a unique pattern of strain YIM M13156^T^ and its four most closely related species. In addition, based on our results and the chromatographic mobility of the polar lipid labeled as phosphatidylmethylethanolamine in the original descriptions of *G. soli* and *G. terrae* by Jin et al. (2013), it is strongly suggested that it was not correctly identified in the original work, since after binding a methyl-group to phosphatidylethanolamine the resultant component would show a higher apolarity and therefore a higher mobility on the plate than phosphatidylethanolamine itself. Hydroxyphosphatidylethanolamine is not known from the outgroup species *Cryptosporangium arvum* (Tamura et al., 1998).

Major fatty acids were the saturated branched-chain iso-C_16:0_ (36.8 ± 1.1%), the monounsaturated C_17:1_ω8c (13.4 ± 0.4%) and the saturated branched-chain iso-C_15:0_ (11.5 ± 0.5%) complemented by iso-C_16:1_ H (5.0 ± 0.4%), C_17:0_ (4.5 ± 0.3%) and C_18:1_9ωc (5.0 ± 0.2%) in agreement with the closest related species (**Table 1**; for an overview of fatty-acid profiles in *Geodermatophilus* see Supplementary Table S2). In addition, the occurrence of 2-hydroxy fatty acids (mainly iso-C_17:0_ 2OH) is also worth mentioning as it supports the presence of OH-PE observed in the polar lipids profiles of strain YIM M13156^T^ and its four most closely related species (for an overview of fatty-acids profiles in *Geodermatophilus* see Supplementary Table S2). The 2-hydroxy fatty acids are a pre-requisite for the synthesis of the hydroxylated polar lipid (Kämpfer et al., 2010).

**FIGURE 4:**
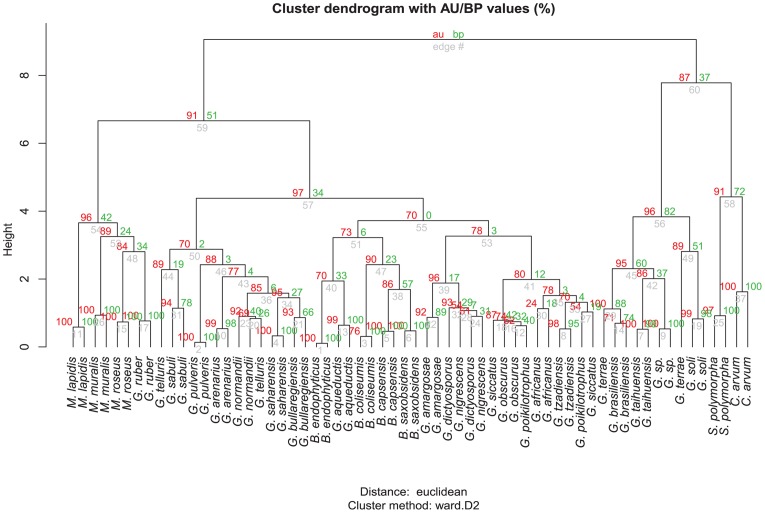
Fatty-acids dendrogram estimated from the logit-transformed percent values as measured with the MIDI system using the Ward algorithm for agglomerative hierarchical clustering and euclidean distances using the *pvclust* package for the *R* statistical environment. Approximately unbiased (AU, left) and bootstrapping (BP, right) support values are shown above the branches.

The clustering analysis of the logit-transformed fatty-acid profiles revealed that those of YIM M13156^T^, *G. brasiliensis, G. soli, G. taihuensis*, and *G. terrae* separated first. Hence the profiles of the other *Geodermatophilus* species were more similar to the ones of *Blastococcus* and *Modestobacter* (**Figure 4**). Accordingly, the *randomForest* analysis identified three minor components (iso-C_17:0_ 10-methyl, C_17:0_ 3OH and iso-C_16:0_ 10-methyl) highly predictive of the group formed by strain YIM M13156^T^, *G. brasiliensis, G. soli, G. taihuensis*, and *G. terrae*. The fatty-acid profiles thus even independently supported the assignment of these five species to a new genus (Supplementary Figures S3, S4).

Whole-cell sugar analysis revealed rhamnose, ribose, mannose, glucose and an unidentified sugar showing a similar chromatographic mobility than the unidentified sugar found in *G. normandii* DSM 45417^T^ by Montero-Calasanz et al. (2013b). On the other hand, *G. soli* DSM 45843^T^, *G. taihuensis* DSM 45962^T^ and *G. terrae* DSM 45844^T^ showed the same sugar profile as *G. brasiliensis* DSM 45426^T^ (Bertazzo et al., 2014), consisting of ribose, mannose, glucose and galactose (Lechevalier and Lechevalier, 1970). The absence of galactose in the profile of strain YIM M13156^T^ might differentiate this species from others within the group.

In order to standardize the phenotypic data available for the genus *Geodermatophilus*, analyses of polar lipids, whole-cell sugars and menaquinones were also carried out for the species *G. obscurus* DSM 43160^T^, *G. ruber* DSM 45317^T^ and *G. nigrescens* DSM 45408^T^. The polar lipid and menaquinone profiles of those species were already specified in the **Table 1** of the original description of *G. arenarius* DSM 45418^T^ by Montero-Calasanz et al. (2012), nevertheless they were never properly described nor the species emended. The three species showed the typical polar-lipid profile observed in *Geodermatophilus* consisting of diphosphatidylglycerol (DPG), phosphatidylethanolamine (PE), phosphatidylcholine (PC), phosphatidylinositol (PI) and minor amounts of phosphatidylglycerol (PG) (see Supplementary Figure S5). Similar to the group formed by *G. brasiliensis* DSM 44526^T^, *G. soli* DSM 45843^T^, *G. taihuensis* DSM 45962^T^ and *G. terrae* DSM 45844^T^, *G. ruber* DSM 45317^T^ displayed glycophosphatidylinositol (GPI) in addition to an unidentified phospholipid (PL). The polar-lipid profiles of *G. obscurus* DSM 43160^T^ and *G. nigrescens* DSM 45408^T^ conversely revealed the typical unidentified glycolipid already described for most *Geodermatophilus* species (See supplementary Table S1). Regarding the MK pattern of *G. obscurus* DSM 43160^T^, apart from MK-9(H_4_) (64.3%) already indicated by Zhang et al. (2011), MK-9(H_2_) (8.7%), MK-9(H_0_) (4.9%) and MK-8(H_4_) (4.3%) were also revealed. Nevertheless, in contrast to Zhang et al. (2011) and in addition to MK-9(H_4_), our studies did not identify MK-9(H_0_) in the profile of *G. ruber* DSM 45317^T^. The whole-cell sugar patterns of *G. obscurus* DSM 43160^T^ revealed the presence of ribose, xylose, mannose, glucose and galactose. *G. ruber* DSM 45317^T^ displayed a profile consisting of ribose and glucose. Differently from Nie et al. (2012) who identified galactose, arabinose and glucosamine as the whole-cell sugar patters of *G. nigrescens* DSM 45408^T^, our results showed a profile comprising mannose, glucose, galactose and traces of rhamnose and ribose. These profiles are consistent with those previously described in the genus, although it is worth mentioning the absence of galactose in the profiles of *G. ruber* DSM 45317^T^, a feature shared, as mentioned previously, with the strain YIM M13156^T^. The presence of xylose was already described for *G. saharensis* DSM 45423^T^ (Montero-Calasanz et al., 2013c).

## Discussion

Phylogenetic analysis based on whole genome and 16S rRNA gene sequences revealed with strong support that strain YIM M13156^T^ and the species *G. brasiliensis* DSM 44526^T^, *G. soli* DSM 45843^T^, *G. taihuensis* DSM 45962^T^ and *G. terrae* DSM 45844^T^ formed a separate lineage within *Geodermatophilaceae*, hence *Geodermatophilus* is not monophyletic. Since the main goal of phylogenetic systematics is to obtain monophyletic taxa (Hennig, 1965; Wiley and Lieberman, 2011) taxonomic consequences are necessary. A lumping approach would require merging all *Geodermatophilaceae* genera into *Geodermatophilus*, which has priority, and thus the generation of 13 new names (i.e., new combinations for *Blastococcus* and *Modestobacter*). In contrast, placing the aberrant *Geodermatophilus* species into a separate genus require the generation of only four new names, one for the new genus and four new combinations. Taxonomic conservatism, most easily be measured as inversely proportional to the number of new names to be created (Breider et al., 2014), thus clearly favors the splitting solution. These two arguments alone justify the need to introduce a new genus of *Geodermatophilaceae*.

Nevertheless, the phenotype also provided rich information on the interrelationships of the envisaged new genus. Strain YIM M13156^T^ and its four neighboring species were distinguished from other genera in the family *Geodermatophilaceae* by cell morphology, the lack of spores, the absence of phosphatidylcholine and the typical unidentified glycolipid found ins *Geodermatophilus*, the presence of glycophosphatidylinositol and hydroxyphosphatidylethanolamine in their polar lipids profiles (for an overview of the characteristics that differentiate the strain YIM M13156^T^, *G. brasiliensis, G. soli, G. taihuensis* and *G. terrae* from closely related *Geodermatophilaceae* genera see Supplementary Table S1 and *randomForest* predictions Supplementary Figure S2) and the occurrence of iso-C_17:0_ 2OH and other minor compounds (Supplementary Figure S4 and Supplementary Table S2) in their fatty-acids patterns.

However, in phylogenetic systematics diagnostic features for a group are insufficient to establish it as a taxon because when these features were plesiomorphic (ancestral) instead of apomorphic (derived) then they could well diagnose a paraphyletic group (Hennig, 1965; Wiley and Lieberman, 2011); reptiles are a classical example. For this reason, we studied the distribution of the above listed features among *Geodermatophilaceae* and the outgroup species *Cryptosporangium arvum* (Tamura et al., 1998) and *Sporichthya polymorpha* (Supplementary Figure S4 and Supplementary Table S2) for determining with maximum-parsimony reconstructions which character state was apomorphic for which group (**Figure 1**). Accordingly, presence of hydroxyphosphatidylethanolamine, iso-C_17:0_ 10-methyl, C_17:0_ 3OH and iso-C_16:0_ 10-methyl appeared as synapomorphies of YIM M13156^T^ and its four neighboring species; presence of glycophosphatidylinositol appeared as autapomorphy of *Geodermatophilaceae*; secondary absence of glycophosphatidylinositol and presence of phosphatidylcholine as synapomorphy of core *Geodermatophilus* and *Blastococcus*; and presence of the unidentified glycolipid as autopomorphies of core *Geodermatophilus*. Hydroxyphosphatidylethanolamine is present in *G. pulveris*, too, but was gained independently; the unidentified glycolipid is missing in *G. ruber* but was secondarily lost (**Figure 1**). The status of cell morphology and spore formation was unclear due to missing data (Supplementary Table S1) but the already assembled evidence clearly supports the envisaged reclassification.

The currently still dominating practice of polyphasic taxonomy (Vandamme et al., 1996) in microbial systematics has increasingly been called into question in recent years (Sutcliffe et al., 2012; Vandamme and Peeters, 2014; Sutcliffe, 2015; Thompson et al., 2015). Critics mainly emphasize that more genomic information should be incorporated and that some of the nowadays routinely conducted phenotypic tests might actually be unnecessary. It was also obvious in the present study that genome-scale data yielded high resolution (**Figure 1**), which via a backbone constraint (Hahnke et al., 2016) could also inform a more comprehensively sampled 16S rRNA gene analysis (**Figure 2**).

Whereas phylogenomics is expected to yield more strongly resolved trees, these might in theory also yield more conflict between distinct analyses (Jeffroy et al., 2006; Klenk and Göker, 2010). Horizontal gene transfer is a known cause of topological conflict between analyses of single genes that has even been be used to argue against hierarchical classification (Bapteste and Boucher, 2009; Klenk and Göker, 2010). However, the increase of support in phylogenomic analyses after adding genes up to virtually all available genes indicates a strong hierarchical signal (Breider et al., 2014), whereas the selection of pre-defined set of few genes does not yield genome-scale data and relies on *a priori* assumptions about the relative suitability of genes for analysis (Lienau and DeSalle, 2009; Klenk and Göker, 2010). Methods such as GBDP, which infer trees rather directly from complete genomes, are more promising for obtaining a truly genome-based classification, but conflict between single genes raises the question of how to not overestimate phylogenetic confidence (Taylor and Piel, 2004). This issue can hardly be overestimated because in phylogenetic systematics taxa must be as well supported as monophyletic as possible (Vences et al., 2013), which rules out all phylogenomic methods that do not even yield statistical support values. Instead of standard bootstrapping, the partition bootstrap, which resamples entire genes, is supposed to reduce conflict and provide more reliable support values (Siddall, 2010; Simon et al., 2017). Within the GBDP pseudo-bootstrapping framework, the greedy-with-trimming algorithm (Meier-Kolthoff et al., 2014a) as applied in the present study is the equivalent of the partition bootstrap (Hahnke et al., 2016).

Properly analyzed genome-scale data thus address the current shortcoming of polyphasic taxonomy that its starting point is an often poorly resolved 16S rRNA gene tree. After choosing taxon boundaries from such a tree, the polyphasic approach would then proceed with determining diagnostic features for the new taxa. Tools such as *randomForest* as used here can assist in the task to select features predictive for a certain group of interest from larger numbers of characters.

However, a more serious problem with the currently dominating polyphasic approach is that such diagnostic features cannot provide independent evidence for taxon boundaries when these boundaries were already used to choose the features. Independent evidence can instead be obtained by detecting the same groups independently when analyzing the additional features (**Figure 3**). Moreover, in phylogenetic systematics diagnostic features are insufficient for justifying a taxon because taxa must be monophyletic, whereas diagnostic character states can be plesiomorphic and thus diagnose a paraphyletic group (Hennig, 1965; Wiley and Lieberman, 2011). To the best of our knowledge, publications applying polyphasic taxonomy hardly ever address these two issues, even though phylogenetic systematics is the appropriate paradigm for microbial taxonomy, too (Klenk and Göker, 2010). Outgroup and ingroup comparisons might sometimes be difficult because of incomplete character sampling but in the present study succeeded in determining that some character states were apomorphies for the envisaged new taxa. We thus believe that microbial taxonomy would not only benefit from incorporating genomic information but also from adhering to the principles of phylogenetic systematics.

### Taxonomic Consequences

Based on phenotypic and genotypic data presented, we propose that strain YIM M13156^T^ represents a novel species of a new genus of *Geodermatophilaceae*, for which the name *Klenkia marina* gen. nov., sp. nov. is proposed. In addition we propose the reclassification of *Geodermatophilus brasiliensis* as *Klenkia brasiliensis* comb. nov., *Geodermatophilus soli* as *Klenkia soli* comb. nov., *Geodermatophilus taihuensis* as *Klenkia taihuensis* comb. nov. and *Geodermatophilus terrae* as *Klenkia terrae* comb. nov. The emendation of the genus *Geodermatophilus* and the species *G. africanus, G. amargosae, G. aquaeductus, G. dictyosporus, G. nigrescens, G. normandii, G. obscurus, G. poikilotrophus, G. pulveris, G. ruber, G. sabuli, G. saharensis, G. siccatus*, and *G. telluris* are also proposed in this study.

### Description of *Klenkia* gen. nov.

Klen’ki.a (N. L. fem. n. *Klenkia*, named in honor of Hans-Peter Klenk, Professor at Newcastle University (United Kingdom) in recognition of his contributions to bacterial systematics including the promotion of studies in *Geodermatophilaceae*).

Cells are motile, rod-shaped and Gram-reaction-positive. The peptidoglycan in the cell-wall contains *meso*-diaminopimelic acid. The predominant menaquinones are MK-9(H_4_) and MK-9(H_0_) but MK-9(H_2_), MK-8(H_4_) and MK-10(H_4_) may also be present in minor amounts. The basic polar-lipids profile includes diphosphatidylglycerol, phosphatidylethanolamine, hydroxyphosphatidylethanolamine, phosphatidylinositol and glycophosphoinositol. In some species an unidentified glycophospholipid may be present. Phosphatidylcholine is absent. Major cellular fatty acids are iso-C_16:0_ and iso-C_15:0_. The basic whole-cell sugar pattern includes ribose, mannose and glucose. The presence of galactose is frequent. Rhamnose may occur in some species. The genomic G+C content is 74.0–75.0%. The type species of *Klenkia* is *Klenkia marina*, sp. nov.

### Description of *Klenkia marina* sp. nov.

K. ma.ri’na. (L. fem. adj. *marina*, of the sea, marine).

Colonies are pink-colored, convex, circular and opaque with a smooth surface and an entire margin. Cells are motile, rod-shaped and Gram-reaction-positive. According to the BIOLOG System: dextrin, D-maltose, D-trehalose, D-cellobiose, sucrose, turanose, β-methyl-D-glucoside, *N*-acetyl-D-glucosamine, D-glucose, D-mannose, D-fructose, D-galactose, inosine,D-mannitol, glycerol, L-alanine, L-glutamic acid, L-pyroglutamic acid, pectin, methyl pyruvate, citric acid, α-keto-glutaric acid, D-malic acid, L-malic acid, bromo-succinic acid, nalidixic acid, lithium chloride, potassium tellurite, α-hydroxy-butyric acid, β-hydroxy-butyric acid, α-keto-butyric acid, acetoacetic acid, propionic acid, acetic acid and aztreonam are positive but stachyose, D-raffinose, alpha-D-lactose, D-melibiose, *N*-acetyl-D-galactosamine, *N*-acetyl-neuraminic acid, 3-*O*-methyl-D-glucose, D-fucose, L-fucose, L-rhamnose, fusidic acid, D-sorbitol, D-arabitol, D-fructose-6-phosphate, D-aspartic acid, D-serine, troleandomycin, rifamycin SV, minocycline, gelatin, L-arginine, L-aspartic acid, L-histidine, lincomycin, guanidinehydrochloride, niaproof, D-galacturonic acid, L-galactonic acid-γ-lactone, D-glucuronic acid, glucuronamide, mucic acid, quinic acid, D-saccharic acid, vancomycin, tetrazolium violet, tetrazolium blue, *p*-hydroxy-phenylacetic acid, L-lactic acid, tween 40, γ-amino-*n*-butyric acid, sodium formate and butyric acid are negative. Cell growth ranges from 20 to 30°C (optimal growth temperature is 25–30°C), from pH 6.0–8.5 (optimal range 6.5–8.0) and 0–4% NaCl. The peptidoglycan in the cell-wall contains *meso*-diaminopimelic acid as diamino acid. The whole-cell sugars are rhamnose, ribose, mannose, glucose and an unidentified sugar. The predominant menaquinones are MK-9(H_4_) and MK-9(H_0_). The main polar lipids are diphosphatidylglycerol, phosphatidylethanolamine, phosphatidylinositol, glycophosphatidylinositol, an unidentified aminolipid and traces of hydroxyphosphatidylethanolamine. Cellular fatty acids consist mainly of iso-C_16:0_, C_17:1_ω9 and iso-C_15:0_. The type strain has a genomic G+C content of 74.4%. The genome size is 4.2 Mbp.

The INSDC accession number for the 16S rRNA gene sequences of the type strain YIM M13156^T^ (=DSM 45722^T^ = CCTCC AB 2012057^T^) is LT746188. The accession number for the whole genome sequence of strain YIM M13156^T^ is FMUH01000001.

### Description of *Klenkia brasiliensis* comb. nov.

K. bra.si.li.en’sis. (N. L. fem. adj. *brasiliensis*, referring to Brazil, the country from where the type strain was isolated).

Basonym: *Geodermatophilus brasiliensis* Bertazzo et al. (2014)

The description is as given by Bertazzo et al. (2014) with the following modification. The genomic G+C content is 74.8%. The genome size is 4.5 Mbp.

The accession number for the whole genome sequence of strain DSM 44526^T^ is FNCF00000000.

The type strain Tü 6233^T^ (= DSM 44526^T^ = CECT 8402^T^) was isolated from soil collected in São José do Rio Preto, São Paulo (20°46′39′′ S, 49°21′35′′ W, altitude 530 m above mean see level), Brazil.

### Description of *Klenkia soli* comb. nov.

K. so’li. (L. gen. n. *soli*, of soil)

Basonym: *Geodermatophilus soli* Jin et al. (2013)

The properties are as given in the species description by Jin et al. (2013) with the following emendation. In addition to diphosphatidylglycerol, phosphatidylethanolamine and phosphatidylinositol, the polar lipids pattern consists in phosphatidylglycerol, hydroxyphosphatidylethanolamine, an unidentified glycophospholipid and glycophosphatidylinositol (the chromatographic mobility of which is documented in Fig. 1b). Phosphatidylcholine and phosphatidylmethylethanolamine are absent. The whole-cell sugars are ribose, mannose, glucose and galactose. MK-9(H_4_) is the predominant menaquinone but also contains MK-9(H_0_) (as listed by Jin et al., 2013) and MK-9(H_2_). The genomic G+C content is 74.2%. The genome size is 4.8 Mbp.

The accession number for the whole genome sequence of strain DSM 45843^T^ is FNIR00000000.

The type strain, PB34^T^ (=DSM 45843^T^ = KCTC 19880^T^ = JCM 17785^T^), was isolated from grass soil in Korea.

### Description of *Klenkia taihuensis* comb. nov.

K. tai.hu.en’sis. (N. L. fem. adj. *taihuensis*, of or pertaining to Taihu Lake, the source of the sediment from which the type strain was isolated).

Basonym: *Geodermatophilus taihuensis* Qu et al. (2013)

The properties are as given in the species description by Qu et al. (2013) with the following emendation. In addition to diphosphatidylglycerol, phosphatidylethanolamine and phosphatidylinositol, the polar lipids pattern consists of phosphatidylglycerol, hydroxyphosphatitylethanolamine and glycophosphatidylinositol (the chromatographic mobility of which is documented in **Figure 1C**). Phosphatidylcholine is absent. The whole-cell sugars are ribose, mannose, glucose and galactose. Meso-diaminopimelic acid is present. MK-9(H_4_) is the predominant menaquinone but the strain also contains MK-9(H_0_), MK-9(H_6_) (as listed by Qu et al., 2013), MK-9(H_2_) and MK-8(H_4_). The genomic G+C content is 74.9%. The genome size is 4.3 Mbp.

The accession number for the whole genome sequence of strain DSM 45962^T^ is FOMD00000000.

The type strain is 3-wff-81T (=DSM 45962^T^ = CGMCC 1.12303^T^ = NBRC 109416^T^), isolated from the superficial sediment of Taihu Lake in Jiangsu Province, China.

### Description of *Klenkia terrae* comb. nov.

K. ter’ra.e. (L. gen. n. *terrae*, of the earth).

Basonym: *Geodermatophilus terrae* Jin et al. (2013)

The properties are as given in the species description by Jin et al. (2013) with the following emendation. In addition to diphosphatidylglycerol, phosphatidylethanolamine and phosphatidylinositol, the polar lipids pattern consists in phosphatidylglycerol, hydroxyphosphatitylethanolamine and glycophosphatidylinositol (the chromatographic mobility of which is documented in **Figure 1D**). Phosphatidylcholine and phosphatidylmethylethanolamine are absent. The whole-cell sugars are ribose, mannose, glucose and galactose. MK-9(H_4_) is the predominant menaquinone but also contains MK-9(H_0_) (as listed by Jin et al., 2013), MK-9(H_2_) and MK-10(H_4_).

The type strain, PB261^T^ (=DSM 45844^T^ = KCTC 19881^T^ = JCM 17786^T^), was isolated from grass soil in Korea.

### Emended Description of the Genus *Geodermatophilus* Luedemann (1968)

The properties are as given by Luedemann (1968) with the following modifications. The peptidoglycan in the whole-cell contains *meso*-diaminopimelic acid. The predominant menaquinone is MK-9(H_4_) but MK-9(H_0_), MK-9(H_2_), MK-9(H_6_), MK-8(H_4_) and MK-10(H_4_) may also be present in significant or minor amounts. The basic polar lipids profile involves diphosphatidylglycerol, phosphatidylethanolamine, phosphatidylcholine, phosphatidylinositol and an unidentified glycolipid. The presence of phosphatidylglycerol is frequent. Major cellular fatty acids are iso-C_16:0_ and iso-C_15:0_. The whole-cell sugar pattern frequently includes ribose, mannose, glucose and galactose as diagnostic sugar. The genomic G+C content is 74.0–76.0%.

### Emended Description of *Geodermatophilus africanus* Montero-Calasanz et al. (2013e)

The properties are as given in the species description by Montero-Calasanz et al. (2013e) with the following modification. The genomic G+C content is 74.3%. The genome size is 5.5 Mbp.

The accession number for the whole genome sequences of the type strain DSM 45422^T^ is FNOT00000000.

### Emended Description of *Geodermatophilus amargosae* Montero-Calasanz et al. (2014a)

The properties are as given in the species description by Montero-Calasanz et al. (2014b) with the following modification. The genomic G+C content is 74.2%. The genome size is 5.9 Mbp.

The accession number for the whole genome sequence of the type strain DSM 46136^T^ is FPBA00000000.

### Emended Description of *Geodermatophilus aquaeductus* Hezbri et al. (2015c)

The properties are as given in the species description by Hezbri et al. (2015c) with the following modification. The genomic G+C content is 75.0%. The genome size is 5.4 Mbp.

The ENA accession numbers for the whole genome sequence of the type strain DSM 46834^T^ are FXTJ01000001-FXTJ01000028.

### Emended Description of *Geodermatophilus dictyosporus* Montero-Calasanz et al. (2015)

The properties are as given in the species description by Montero-Calasanz et al. (2015) with the following modification. The DNA G+C content is 75.3% (genome sequence). The genome size is 5.0 Mbp.

The ENA accession numbers for the whole genome sequence of the type strain DSM 43161^T^ are FOWE01000001-FOWE01000022.

### Emended Description of *Geodermatophilus nigrescens* Nie et al. (2012)

The properties are as given in the species description by Nie et al. (2012) with the following emendation. It grows well on GYM and GPHF media but poor on TSA and not on R2A, GEO, Luedemann and PYGV media. The following enzymatic activities according to API ZYM strips are present: alkaline phosphatase, esterase lipase, leucine arylamidase, valine arylamidase and α-glucosidase. In addition to diphosphati dylglycerol, phosphatidylglycerol, phosphatidylethanolamine and phosphatidylcholine the polar lipids profile contains phosphatidylinositol and two unidentified glycolipids (the chromatographic mobility of which is documented in Supplementary Figure S5A). The whole-cell sugars are mannose, glucose, galactose and traces of rhamnose and ribose. Arabinose and glucosamine as listed by Nie et al. (2012) are absent. The genomic G+C content is 75.9%. The genome size is 4.7 Mbp.

The accession number for the whole genome sequences of strain DSM 45408^T^ is FQVX00000000.

### Emended Description of *Geodermatophilus normandii* Montero-Calasanz et al. (2013b)

The properties are as given in the species description by Montero-Calasanz et al. (2013b) with the following modification. The DNA G+C content is 75.3% (genome sequence). The genome size is 4.6 Mbp.

The IMG accession number for the whole genome sequence of the type strain DSM 45417^T^ is 2585427554.

### Emended Description of *Geodermatophilus obscurus* Luedemann (1968)

The properties are as given in the species description by Luedemann (1968) with the following emendation. The temperature range is from 15.0°C to 40.0 with an optimum range from 28°C to 37°C. pH range is 6.0–9.0 (optimal range 6.5–8.5). It grows well on GYM and regular on GPHF media but not on R2A, GEO, TSA, Luedemann and PYGV media. It degrades starch but not tyrosine, xanthine, casein, hypoxanthine. The following enzymatic activities according to API ZYM strips are present: alkaline phosphatase, esterase lipase, leucine arylamidase. Catalase positive but oxidase negative. Hydrolisis of aesculin and gelatine. The polar lipids profile consists in diphosphatidylglycerol, phosphatidylglycerol, phosphatidylethanolamine, phosphatidylcholine, phosphatidy linositol and unidentified glycolipid (the chromatographic mobility of which is documented in Supplementary Figure S5B). Whole-cell sugars are ribose, xylose, mannose, glucose and galactose. *Meso*-diaminopimelic acid is present in the cell-wall. MK-9(H_4_) is the predominant menaquinone, but MK-9(H_2_), MK-9(H_0_) and MK-8(H_4_) are present as minor components. The genome size is 5.3 Mbp. The genomic G+C content is 73.9% (Ivanova et al., 2010).

### Emended Description of *Geodermatophilus poikilotrophus* corrig. Montero-Calasanz et al. (2015)

The properties are as given in the species description by Montero-Calasanz et al. (2014) with the following modification. The genomic G+C content is 74.6%. The genome size is 4.8 Mbp.

The accession number for the whole genome sequence of the type strain DSM 44209^T^ is FOIE00000000.

### Emended Description of *Geodermatophilus pulveris* Hezbri et al. (2016a)

The properties are as given in the species description by Hezbri et al. (2016a) with the following modification. The genomic G+C content is 75.6%. The genome size is 4.4 Mbp.

The accession number for the whole genome sequence of the type strain DSM 46839^T^ is FZOO00000000.

### Emended Description of *Geodermatophilus ruber* Zhang et al. (2011)

The properties are as given in the species description by Zhang et al. (2011) with the following emendation. It grows well on GYM, TSA and R2A media but not on GEO, Luedemann, PYGV and GPHF media. In addition to diphosphatidylglycerol, phosphatidylethanolamine, phosphatidylinositol and two unidentified phospholipids, the polar lipids pattern consists in phosphatidylglycerol, phosphatidylcholine and an unidentified glycolipid (the chromatographic mobility of which is documented in Supplementary Figure S5C). The whole-cell sugars are ribose and glucose. Oxidase and catalase positive. Aesculin hydrolysis present. MK-9(H_4_) is the predominant menaquinone. MK-9(H_0_) as listed by Zhang et al. (2011) is absent. The genomic G+C content is 74.0%. The genome size is 5.0 Mbp.

The accession number for the whole genome sequence of strain DSM 45317^T^ is FOSW00000000.

### Emended Description of *Geodermatophilus sabuli* Hezbri et al. (2015a)

The properties are as given in the species description by Hezbri et al. (2015a) with the following modification. The genomic G+C content is 74.0%. The genome size is 5.5 Mbp.

The accession number for the whole genome sequence of the type strain DSM 46844^T^ is OBDO00000000.

### Emended Description of *Geodermatophilus saharensis* Montero-Calasanz et al. (2013c)

The properties are as given in the species description by Montero-Calasanz et al. (2013c) with the following modification. The genomic G+C content is 75.6%. The genome size is 4.9 Mbp.

The accession number for the whole genome sequence of the type strain DSM 45423^T^ is FZOH00000000.

### Emended Description of *Geodermatophilus siccatus* Montero-Calasanz et al. (2013f)

The properties are as given in the species description by Montero-Calasanz et al. (2013f) with the following modification. The genomic G+C content is 74.6%. The genome size is 5.2 Mbp.

The accession number for the whole genome sequence of the type strain DSM 45419^T^ is FNHE00000000.

### Emended Description of *Geodermatophilus telluris* Montero-Calasanz et al. (2013d)

The properties are as given in the species description by Montero-Calasanz et al. (2013d) with the following modification. The genomic G+C content is 75.7%. The genome size is 4.8 Mbp.

The accession number for the whole genome sequences of the type strain DSM 45421^T^ is FMZF00000000.

## Author Contributions

MCM-C, W-JL, and MG designed the study. MCM-C, D-FZ, AY, MR, and PS performed experiments. JM-K and MG performed bioinformatics analysis. MG, TW, and NK sequenced genomes. MCM-C, PS, and MG wrote the manuscript. All authors read and approved the manuscript.

## Conflict of Interest Statement

The authors declare that the research was conducted in the absence of any commercial or financial relationships that could be construed as a potential conflict of interest.
