# Supplementary material for: Genome-Scale Data Call for a Taxonomic Rearrangement of Geodermatophilaceae
Source: Front Microbiol. 2017 Dec 19;8:2501. doi: 10.3389/fmicb.2017.02501 (PMC5742155; doi:10.3389/fmicb.2017.02501)
Supplement: Supplementary file 3 [file Presentation_1.pdf]

Genome-scale data call for a taxonomic rearrangement of *Geodermatophilaceae*

Maria del Carmen Montero-Calasanz[1,2]*, Jan P. Meier-Kolthoff[2], Dao-Feng Zhang[3], Adnan Yaramis[1,4], Manfred Rohde[5], Tanja Woyke[6], Nikos C. Kyrpides[6], Peter Schumann[2], Wen-Jun Li[3*], Markus Göker[2*]

[1]School of Biology, Newcastle University, Ridley Building, Newcastle upon Tyne, NE1 7RU, United Kingdom.
[2]Leibniz Institute DSMZ – German Collection of Microorganisms and Cell Cultures, Inhoffenstraße 7B, 38124 Braunschweig, Germany.
[3]State Key Laboratory of Biocontrol and Guangdong Provincial Key Laboratory of Plant Resources, School of Life Sciences, Sun Yat-Sen University, Guangzhou, 510275, China.
[4]Department of Biotechnology, Middle East Technical University, 06800 Ankara, Turkey.
[5]Central Facility for Microscopy, HZI – Helmholtz Centre for Infection Research Inhoffenstraße 7, 38124 Braunschweig, Germany
[6]Department of Energy Joint Genome Institute, Walnut Creek, CA, USA.

*Corresponding authors:
maria.montero-calasanz@ncl.ac.uk, Tel: +44 (0) 191 208 4943
liwenjun3@mail.sysu.edu.cn
markus.goeker@dsmz.de

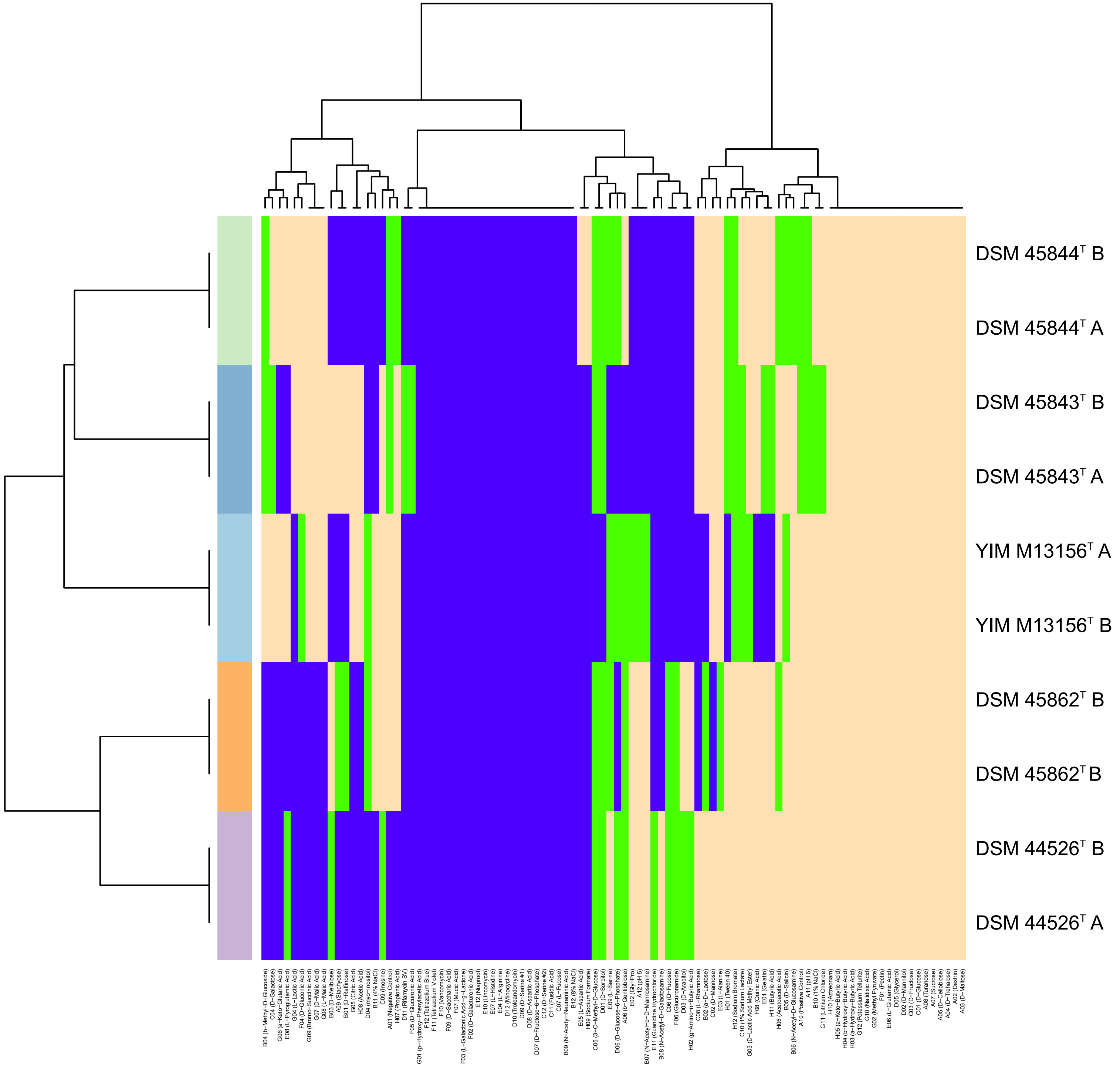

Supplementary Fig. S1. Heatmap showing the parameter "Maximum Height" estimated from the respiration curves as measured with the OmniLog phenotyping device. Plates and substrates are rearranged according to their overall similarity (as depicted using the row and column dendrograms). Ochre colour indicates positive reaction; purple colour indicates negative reaction; green colour indicates ambiguous reaction. Letters (A/B) indicate each replicate of experiment.

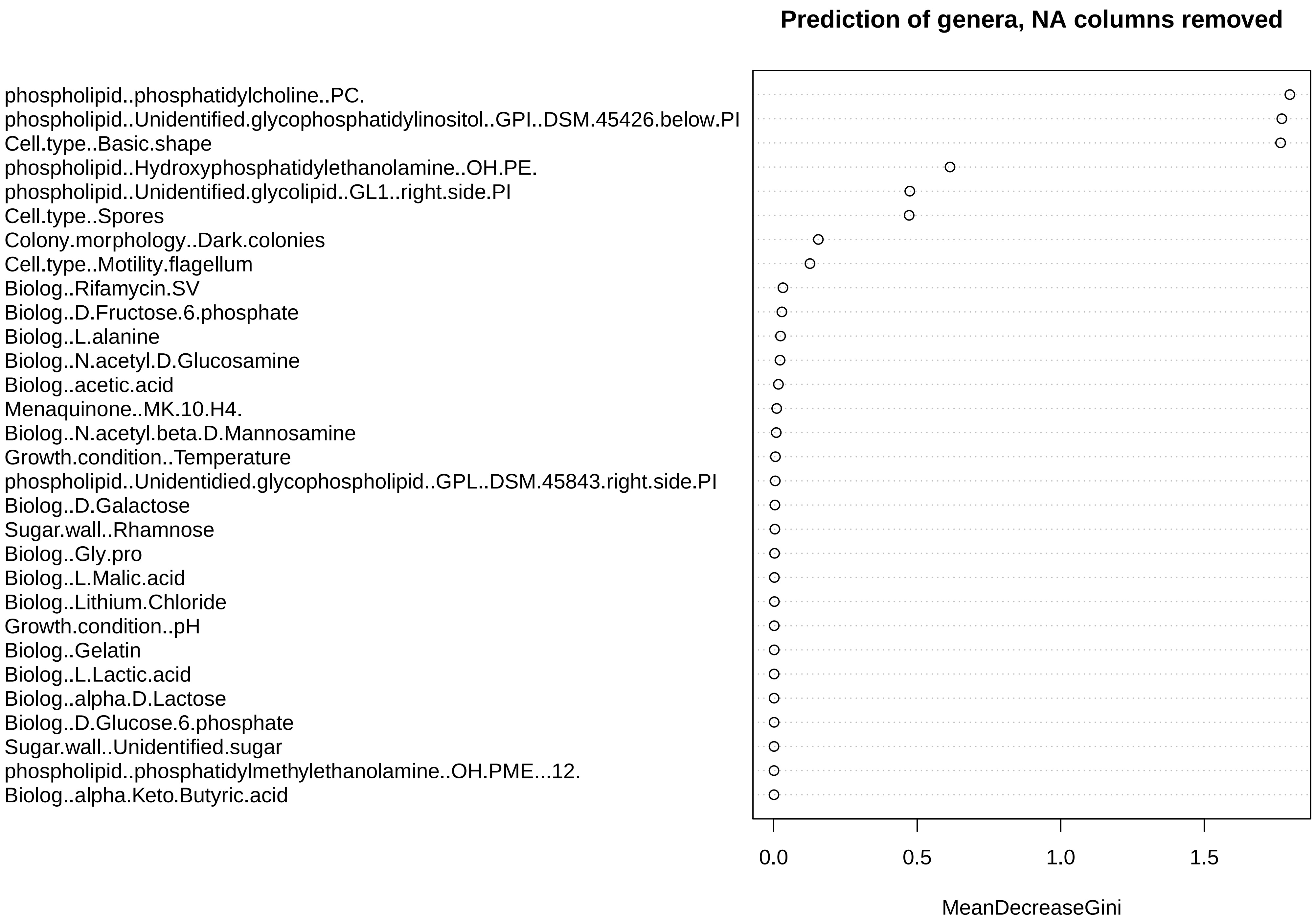

Supplementary Fig. S2. Random forest analysis for predicting the affiliation to the new genera from phenotypic features for all species of interest.

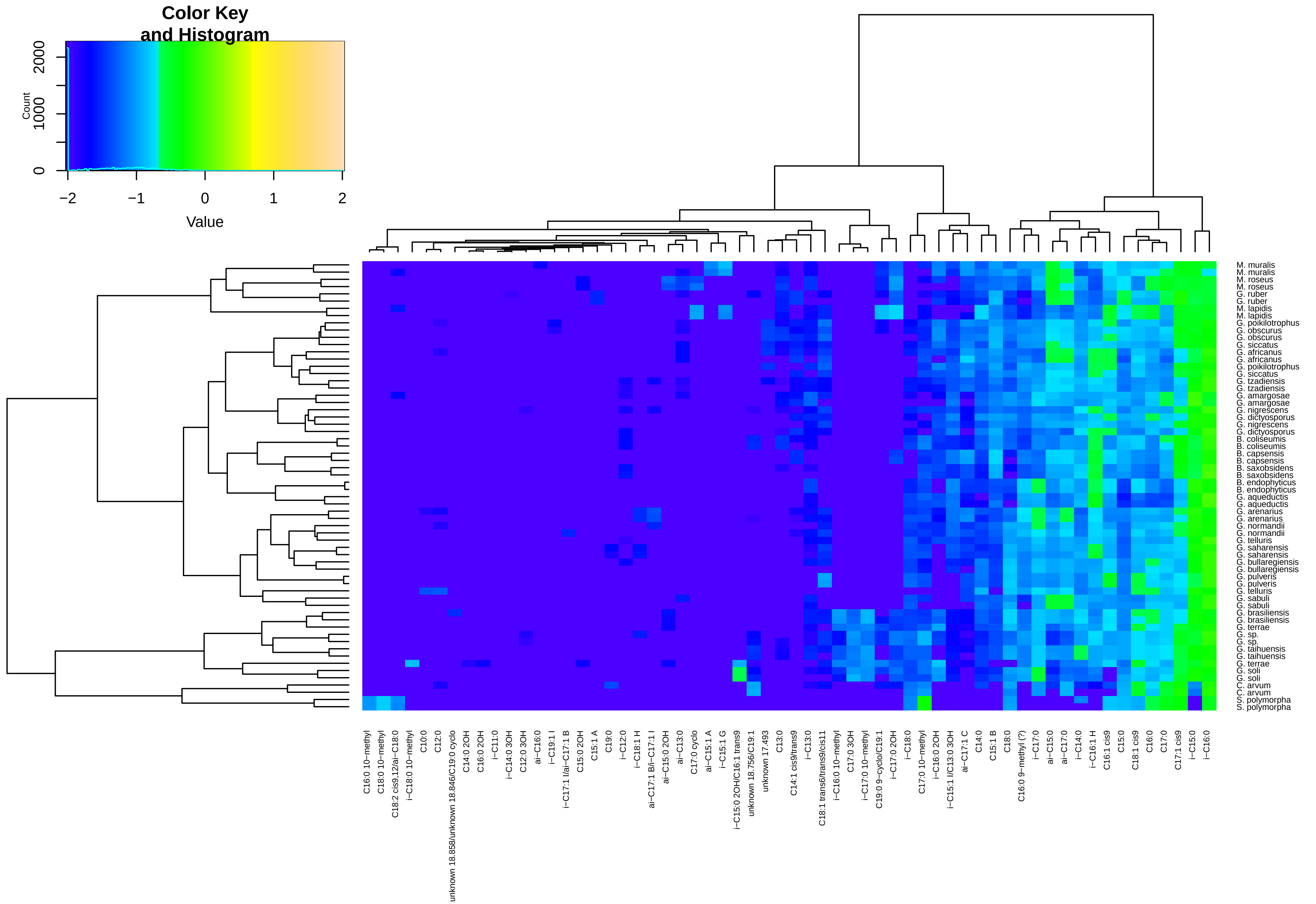

Supplementary Fig. S3 Heatmap showing the percent values as measured with the MIDI system. Samples and fatty acids are rearranged according to their overall similarity (as depicted using the row and column dendrograms).

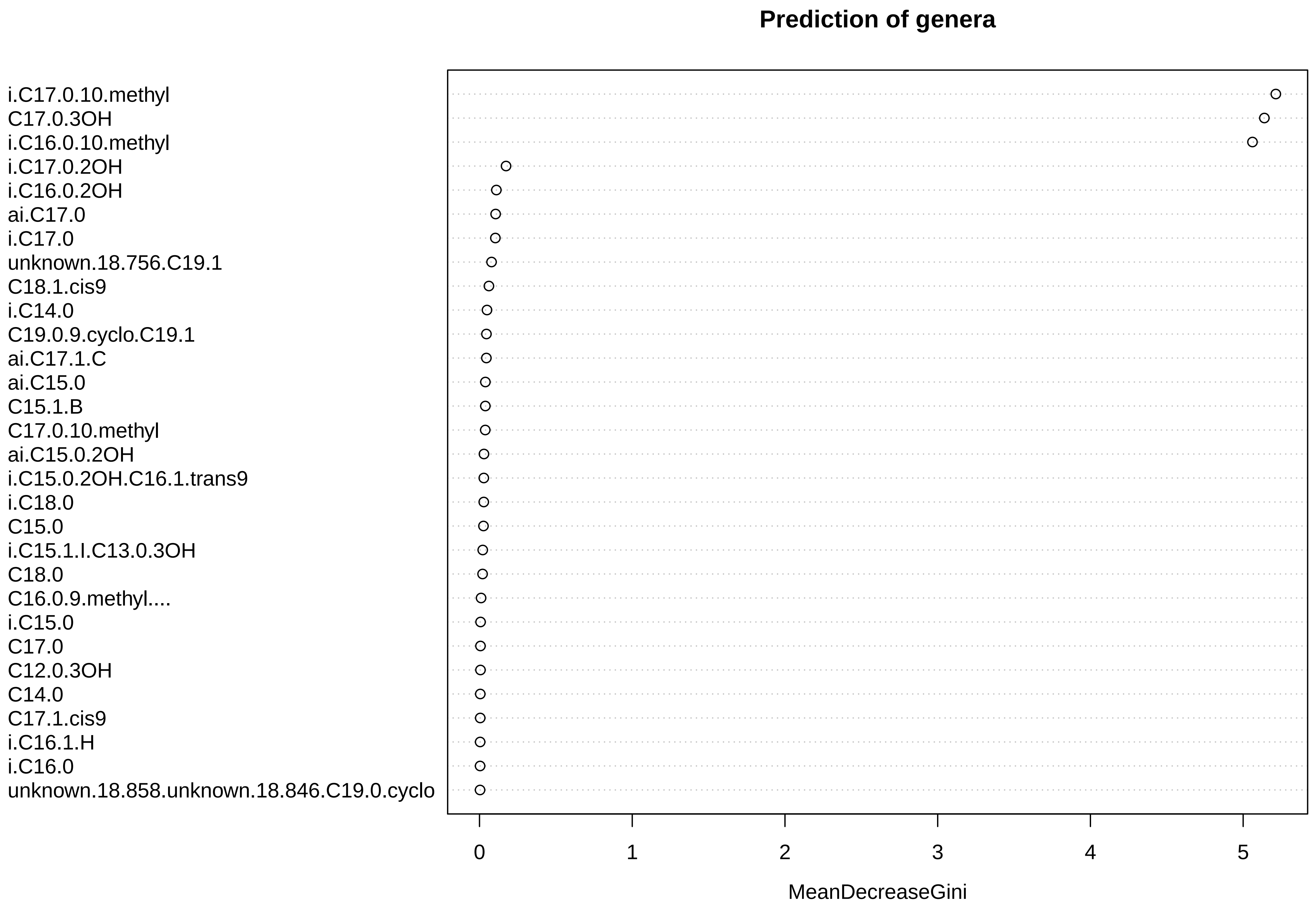

Supplementary Fig. S4. Random forest analysis for predicting the affiliation to the new genera from fatty acids profiles as identifed by MIDI System for all species of interest.

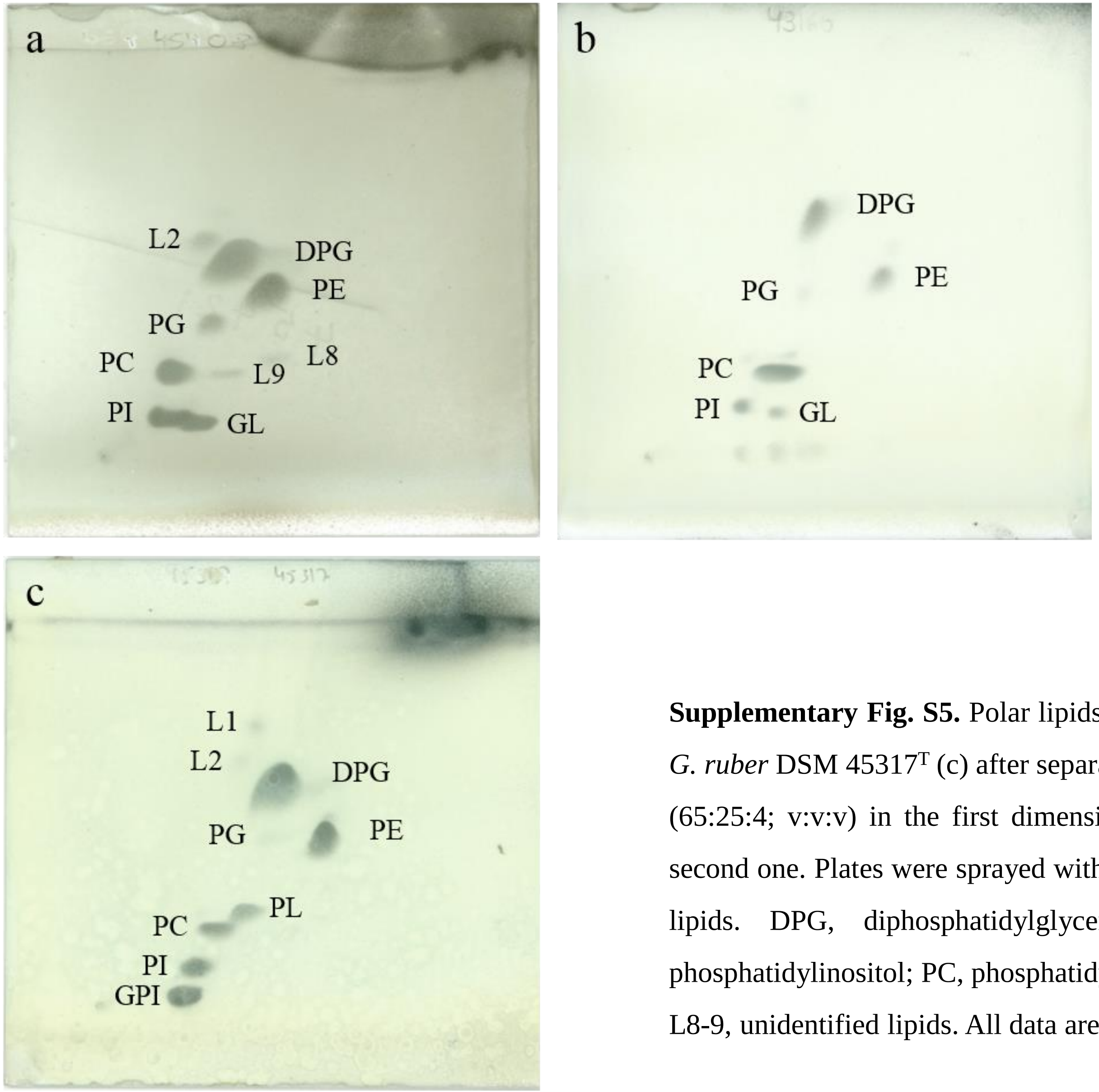

Supplementary Fig. S5. Polar lipids profile of *G. nigrescens* DSM 45408^{T} (a), *G. obscurus* DSM 43160^{T} (b) and *G. ruber* DSM 45317^{T} (c) after separation by two-dimensional TLC using the solvents chloroform:methanol:water (65:25:4; v:v:v) in the first dimension and chloroform:methanol:acetic acid:water (80:12:15:4; v:v:v:v) in the second one. Plates were sprayed with molybdatophosphoric acid (3.5 %; Merck^{TM}) for detection of the total polar lipids. DPG, diphosphatidylglycerol; PG, phosphatidylglycerol; PE, phosphatidylethanolamine; PI, phosphatidylinositol; PC, phosphatidylcholine; GPI, glycophosphatidylinositol; GL, unidentified glycolipid; L1-2, L8-9, unidentified lipids. All data are from this study.
